# Shiga toxin-producing *Escherichia coli* illness in Aotearoa | New Zealand, 2016-2022: epidemiological, genomic and traditional typing analyses provide insight into a significant endemic disease while highlighting knowledge gaps

**DOI:** 10.3389/fmicb.2025.1605469

**Published:** 2025-07-02

**Authors:** Jacqueline Wright, David Duncan, Hugo Strydom, Shevaun Paine, Sarah Jefferies, Joep de Ligt, Lucia Rivas, Michael Addidle, Adrian L. Cookson, David Winter, Hilary Miller, Geraldine Casey, Jing Wang

**Affiliations:** ^1^Institute of Environmental Science and Research Limited (ESR), Christchurch Science Centre, Christchurch, New Zealand; ^2^ESR, Wallaceville Science Centre, Upper Hutt, New Zealand; ^3^ESR, Kenepuru Science Centre, Porirua, New Zealand; ^4^Hartwig Medical Foundation, Amsterdam, Netherlands; ^5^Pathlab Waikato, Tauranga, New Zealand; ^6^Tāwharau Ora—School of Veterinary Science, Massey University, Palmerston North, New Zealand; ^7^AgResearch, Hopkirk Research Institute, Palmerston North, New Zealand; ^8^Department of Public Health HSE Midwest, Limerick, Ireland

**Keywords:** Shiga toxin-producing *Escherichia coli*, STEC, surveillance, epidemiology, epidemiological typing, whole genome sequencing, seropathotype, severity

## Abstract

**Introduction:**

Shiga toxin producing *Escherichia coli* (STEC) cause significant endemic disease in Aotearoa | New Zealand (NZ) with a 2022 case incidence rate of 19.9/100,000 population. The introduction of culture independent diagnostic testing has been pivotal in elucidating STEC case numbers.

**Methods:**

Epidemiological data from 5,769 cases of STEC infection confirmed during the 7-year period 2016-2022 were reviewed in conjunction with epidemiological typing data from 3,746 case isolates (2,939 analyzed via whole genome sequencing).

**Results and discussion:**

Severe illness was reported for 25% of all STEC cases, and 23% of cases were hospitalized. All age groups were affected, but the greatest level of morbidity was observed in those less than 5 years old where the hemolytic uremic syndrome (HUS) incidence rate was 2.86/100,000. Serotypes O157:H7 and O26:H11 together accounted for 54% of infections. Other common serotypes differed from those reported as common elsewhere and included O128:H2, O38:H26, O146:H21 and O91:H14. Shiga toxin subtype 2a strains were more associated with serious illness than other subtypes, regardless of *eae* positivity. Multiple STEC strains were inadvertently identified in 1.2% of culture positive case samples suggesting that carriage of more than one strain could be more prevalent. Single nucleotide polymorphism (SNP) analysis indicated that most STEC cases were sporadic as 5-SNP genomic clusters were uncommon. Infection sources are rarely proven as food and environmental sample data are limited and insufficient to assist in determining pathways to infection. By combining isolate WGS-derived typing data and case epidemiological data we demonstrated the importance of shifting the focus from a select number of STEC serogroups to composite seropathotypes—based on full serotype, ST, cgMLST relatedness and *stx* sub-toxin profile—to assist in understanding both the role of each type in disease severity, and relationships across historical type groups. This knowledge may be useful in future prioritizing of clinical and public health resources.

## 1 Introduction

Shiga toxin-producing *Escherichia* (*E.*) *coli* (STEC) are a heterogeneous group of *E. coli* that are linked by their ability to produce Shiga toxins (Stx) and cause a wide spectrum of clinical syndromes; including mild to severe acute gastroenteritis (AG), hemorrhagic colitis (HC), and hemolytic uremic syndrome (HUS) (Jenkins et al., 2020; Liu et al., 2021; Holtz and Tarr, 2023). HUS can lead to permanent physical and psychological disabilities, and death (3% among children and up to 20% in older age groups) (Freedman et al., 2023).

In Aotearoa | New Zealand (NZ), a south pacific island nation with a population of 5.1 million (Statistics New Zealand, 2024), STEC was initially notifiable under Acute Gastroenteritis (AG) but became notifiable as a separate entity in 2012 (New Zealand Parliamentary Council, 2012). Here, a confirmed case of STEC is defined as a clinically compatible illness (AG or HUS or Thrombotic Thrombocytopenic Purpura), accompanied by laboratory definitive evidence by either culture-based isolation of STEC, or detection of the genes (*stx1* and/or *stx2*) associated with the production of Stx (Health New Zealand Te Whatu Ora, 2012b). Laboratory detections are directly notified to the Public Health Service (PHS), who then investigate and administer a standardized questionnaire (case report form) to confirmed cases for surveillance purposes (ESR, 2023). Case and laboratory data are recorded in the national notifiable diseases database (EpiSurv) administered by the Institute of Environmental Science and Research Limited (ESR) on behalf of the NZ Ministry of Health.

The first laboratory confirmed STEC O157:H7 case in NZ was reported in 1993 (Wright et al., 1993). Following that first case report, diagnostic laboratories in NZ performed culture isolation of *E. coli* O157:H7 using sorbitol MacConkey agar supplemented with cefixime and potassium tellurite (CT-SMAC) on fecal specimens from patients who met certain epidemiological or clinical criteria. e.g., aged less than 5 years, presence of HUS or bloody diarrhea (Rivas et al., 2024). Non-O157 STEC were rarely detected on CT-SMAC from AG fecal specimens, and those that were, such as *E. coli* O84:H2 often had the same sorbitol non-fermenting, CT-resistant phenotypic characteristics as O157:H7 (Cookson et al., 2006a).

In 2015, the largest diagnostic laboratory in NZ (which serves more than 20% of the NZ population) introduced routine culture independent diagnostic testing (CIDT) using commercial nucleic acid amplification technology [EntericBio panel (Serosep, Ireland)] to detect a suite of enteric pathogens, including STEC in all fecal samples referred for testing (McAuliffe et al., 2017). Other diagnostic laboratories across NZ have since transitioned to using CIDT platforms, and by the end of 2022 more than 85% of all patient samples were being screened by CIDT via testing panels from AusDiagnostics (Australia); BDMAX™, BD Diagnostics (Maryland, United States); BioFire™ FilmArray™ (bioMérieux Utah, United States); as well as EntericBio. The last diagnostic laboratory in NZ to transition to CIDT for enteric pathogens did so in March 2024.

Here, we present both the descriptive and typing epidemiology of NZ STEC infections from 2016 to 2022. The time period was selected to include the bulk of the CIDT transition. The aim of this study was to better understand the extent and health impact of STEC disease; the role of various seropathotypes in disease severity; and to examine the added value of whole genome sequencing (WGS) analysis in STEC surveillance; with the overarching intent of improving real time surveillance of STEC infection leading to better health outcomes in NZ.

## 2 Materials and methods

### 2.1 Epidemiological methods

Case report form data from confirmed STEC cases notified 1 January 2016-31 December 2022 were extracted from the EpiSurv database on 24 February 2023 and analyzed for annual incidence, and the following quantitative and qualitative variables with the Statistics New Zealand mid-year general population estimates population as a reference (Statistics New Zealand, 2024): case age, sex, disease severity (symptoms, hospitalization and death), seasonality, travel history, ethnicity, health district and rurality (Environmental Health Intelligence New Zealand, 2024; Statistics New Zealand, 2024). Severe illness was defined as cases with HC, and/or TTP, and/or HUS; and for cases recorded as more than one of these, the case’s severity was counted once as the most severe presentation. Ethnicity numbers were based on prioritized classification of self-determined ethnicity grouped in order of Māori, Pacific peoples, Asian, Middle Eastern/Latin American/African (MELAA) and European or Other (including New Zealander) (Health New Zealand Te Whatu Ora, 2017).

Analyses were performed using Microsoft Excel and R version 4.4.0 using Tidyverse (Wickham et al., 2019; R Core Team, 2024).

### 2.2 Laboratory and typing methods

#### 2.2.1 Microbiology

Diagnostic laboratories routinely culture fecal samples that are *stx* positive (*stx* +) by CIDT onto two selective media: CHROMagar™ STEC and blood agar with vancomycin, cefixime, and cefsulodin (BVCC); and refer these cultures to the national Enteric Reference Laboratory at ESR for testing (Health New Zealand Te Whatu Ora, 2012a; Rivas et al., 2024). Twelve colonies (six from each culture plate referred from a case’s fecal sample) are screened for *stx* + organisms using a multiplex PCR method (Paton and Paton, 1998). Organisms which are *eae* positive but *stx* negative by this method are then further screened for *stx2f* using a conventional PCR (Scheutz et al., 2012).

For the period January 2016-August 2019, all STEC case isolates confirmed at ESR were serotyped by phenotypic serotyping (Ørskov et al., 1984) and tested for *stx1*, *stx2*, *eae*, and *ehxA* using PCR (Paton and Paton, 1998). During this time, a selection of isolates was also analyzed via WGS analysis as part the initialization and validation of ESR’s WGS methodology.

#### 2.2.2 Genomics

From August 2019, all STEC isolates were routinely analyzed via the following WGS methods: Genomic DNA extracted with either the Chemagic™ 360 extraction platform (PerkinElmer, Waltham, MA, United States) or the DNeasy Blood and Tissue Kit (Qiagen, Hilden, Germany); libraries were prepared using either the Illumina Nextera XT library kit (Illumina, San Diego, CA, United States) or the plexWell96 library kit (seqWell, Beverly, MA, United States) for 150 bp pair-end sequencing using either the Illumina NextSeq or Miseq platform (Illumina, San Diego, CA, United States). Sequencing read files were initially analyzed using an in-house quality control pipeline comprising read quality checking, *de novo* assembly and species identification using open-source tools Fastp v.0.20.1 (Chen et al., 2018), Centrifuge v.1.0.4 (Kim et al., 2016), Skesa v. 2.3.0 (Souvorov et al., 2018) and Quast v.5.0.2 (Gurevich et al., 2013). MLST v.2.19 (Seemann et al., 2015) referencing the PubMLST database (Jolley et al., 2018) was used for seven-gene multi-locus sequence type (MLST) assignment (Wirth et al., 2006).

Isolates that passed pre-set quality parameters including contigs < 400 and depth > 45 were subsequently analyzed using the in-house WGS typing pipeline. This pipeline included a combination of mapping [SRST2 v.0.2.0 (Inouye et al., 2014)] and assembly-based [ABRicate v.1.0.1 (Seemann, 2015)] approaches to infer serotype and seven-gene MLST (ST); and to identify virulence genes including *stx* subtype, *aggR*, *aaiC* (encoding for the aggregative adherence fimbriae associated with enteroaggregative *E. coli*—EAEC); *lt* (heat labile toxin associated with Enterotoxigenic *E. coli*—ETEC); *st* (heat stable toxin associated with ETEC); *bfpA* (encoding for bundle-forming pilus associated with enteropathogenic *E. coli*—EPEC); and *ipaH* (encoding for the invasin associated with enteroinvasive *E. coli*—EIEC) (Geurtsen et al., 2022). This enabled recognition of any STEC exhibiting hybrid pathogenicity (genes characteristic of more than one *E. coli* pathogenicity type) (Rodwell et al., 2025).

Both tools referenced Virulence Factor Database VFDB (Chen et al., 2016) and MLST (Seemann et al., 2015); whilst ABRicate alone referenced the SerotypeFinder database (Joensen et al., 2015); and SRST2 referenced EcOH (Ingle et al., 2016).

If an isolate yielded a ST that was novel to the pipeline tools (a novel locus sequence or combination of loci), the genomic reads were submitted to EnteroBase (Zhou et al., 2020) for assignment of a new ST.

#### 2.2.3 Clustering

For the years 2016 and 2017, pulsed field gel electrophoresis (PFGE) analysis of all STEC O157:H7 case isolates was carried out using the standard PulseNet protocol (Pulsenet International, 2013) using *Xba*I restriction enzyme. On occasion additional analysis was performed using *Bln*I to give greater differentiation between strains. The restriction digestion patterns obtained were loaded into BioNumerics 7.62 (Applied Maths, Kortrijk, Belgium). Similarities between pulsotypes were calculated using the Dice coefficient with band matching parameters of 0.5% optimization and 1.5% position tolerance. Inter-strain relationships were assessed by cluster analysis using the Unweighted Pair-Group with Mathematical Average (UPGMA) method. Pulsotypes were locally assigned based on BioNumerics marking the isolates as 100% similarity, but these results were modified, as necessary following visual inspection of the PFGE pattern.

Fine clustering serotype-specific single nucleotide polymorphism (SNP) analysis was performed weekly on all STEC O157:H7 and O26:H11 isolates from August 2019 onward using two tools: SnapperDB v.1.0.6 (Dallman et al., 2018) and Snippy v.4.3.6 (Seemann et al., 2020) and internationally accepted serotype specific reference genomes: the Sakai reference genome (GenBank accession BA000007) for STEC O157:H7 (Hayashi et al., 2001), and 11368 (GenBank accession NC_013361.1) for O26:H11 (Ogura et al., 2009), with each tool separately mapping each genome to the reference strain. Case isolates within five SNP differences by Snapper DB SNP address (using single linkage clustering) were considered to have been potentially exposed to a common source and were assigned a 5-SNP “ClusterID” based on the year of first isolation and its chronological place in the number of clusters identified that year. A case isolate that did not cluster within five SNP differences of any other isolate within the dataset by the Snapper DB SNP address was called a singleton. Maximum likelihood trees were built from the core genome SNP alignments with IQ-tree version 2.0.6 (Nguyen et al., 2014) using built-in model selection and 2000 bootstrap replicates, and visualized in Microreact (Argimón et al., 2016) and Interactive Tree of Life (iTOL) v7.0 (Letunic and Bork, 2024).

Assembly-based allele calls were performed on all STEC genomes using chewBBACA v.3.2.0 (Silva et al., 2018) referencing a core genome MLST (cgMLST) scheme (PubMLST, 2023) which recognizes 2,513 loci within the *E. coli* core genome. The distance matrix from cgMLST allele calling was calculated by cgmlst-dists v.0.4.0 (Seemann, 2020). Core-genome MLST differences were visualized using minimum spanning trees generated by GrapeTree v.2.1 (Zhou et al., 2018). Tree proximity in conjunction with the cgMLST distance matrix were used to inform the broad relatedness of various STEC serotypes. Broad clusters (proximity of < 1,000 differences) were compared to EnteroBase (Zhou et al., 2020; Achtman et al., 2022; Dyer et al., 2024) ST Complex and HC1100 cgST Complex designations before being assigned an ST Group based on proximity of < 1,000 differences. CgMLST was not used for fine cluster determination in this study.

## 3 Results

### 3.1 Descriptive epidemiology of human STEC illness in New Zealand 2016-2022

**FIGURE 1:**
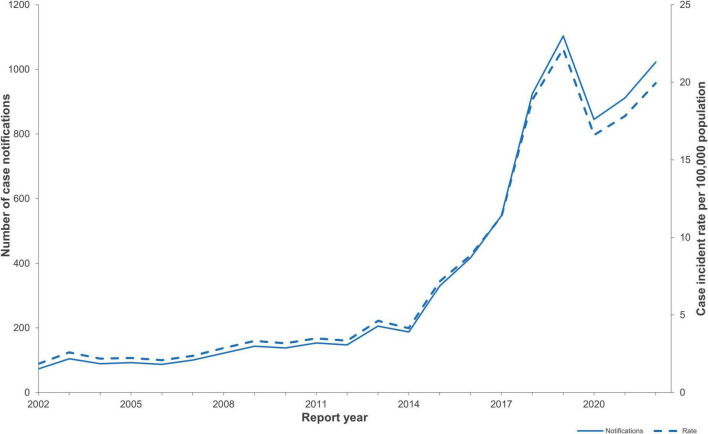
NZ STEC case notification numbers and case incidence rates/100,000 population for the years 2002-2022 (EpiSurv extract 24th February 2023).

**FIGURE 2:**
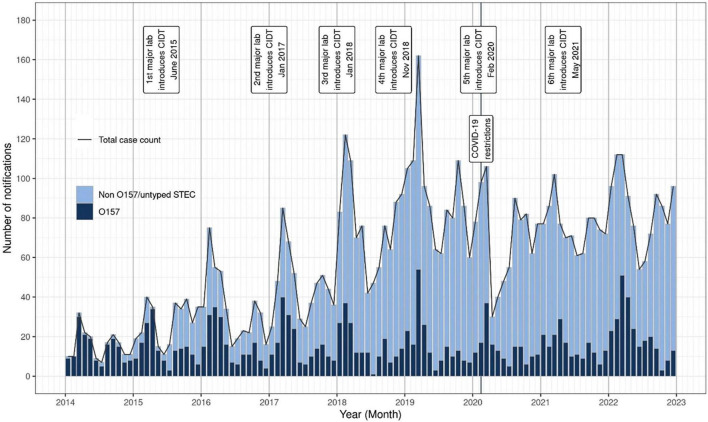
NZ STEC notification numbers by month and year, January 2014-December 2022. The date of introduction of culture-independent detection methods (CIDT) by six main diagnostic laboratories is marked, as is the date of introduction of national COVID-19 protection measures.

Between 2016 and 2022, 5,769 notified cases of STEC were confirmed in NZ. The number of notified cases and associated incidence rates increased year on year from 2015 (417 cases, incidence rate 8.8/100,000 population) onward and peaked at 1,103 cases (incidence rate of 22.4/100,000 population) in 2019 (Figure 1). The increase in case numbers from 2015 coincided with the progressive introduction of CIDT for the testing of fecal samples in NZ in diagnostic laboratories (Figure 2). The ability of CIDT to detect non-O157 STEC, together with the increased screening of a wider sample population resulted in a substantial increase in the number of non-O157 STEC cases from 2015. During the 4-year period 2016-2019, a travel history was recorded for 90.7% (*n* = 2,714) of NZ STEC cases, of these 11.9% (*n* = 322) of cases had a history of overseas travel. During the subsequent 3-year period (2020-2022), a travel history was recorded for 77.1% (*n* = 2143) of cases, of these 2.6% (*n* = 56) had a recent history of overseas travel.

Annual notified case numbers and incidence rates decreased in 2020 to 845 cases and 16.6/100,000 beginning with a marked decrease of notified STEC cases from late March-May 2020 coinciding with the emergence of the COVID-19 pandemic and the implementation of associated national protection measures (Figures 1, 2). Case numbers and incidence rates increased in 2021 (911 cases, and 17.8/100,000) and again in 2022 (1,022 cases, 19.9/100,000).

**FIGURE 3:**
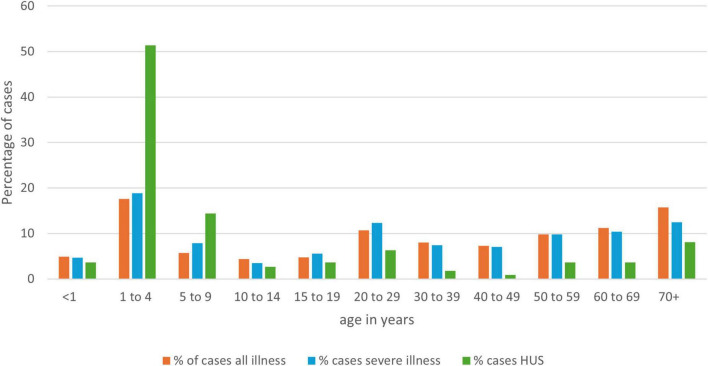
Age distribution of NZ STEC cases as a percentage of all cases, by disease severity for the years 2016-2022 (EpiSurv extract 24th February 2023).

Most (74.7%) of the 5,769 cases confirmed in the 7-year period presented with AG, but 1,459 cases (25.3%) presented with severe illness: HC, 1329 cases (23.0%); HUS 111 cases (1.9%); TTP, 19 cases (0.3%). A case reported as having more than one of these severe presentations was only counted once with preference to the most severe category. Illness was distributed across all age groups (Figure 3), with the median age for all STEC cases being 33 years; for cases of severe disease, 28 years; and for HUS cases, 3 years. Fifty-five percent (61/111) of HUS cases were aged under 5 years, with an averaged HUS incidence rate of 2.86 cases/100,000 for this age group.

Of the 5,769 cases, 5,431 cases (94.1%) had hospitalization data recorded as yes/no, and 1,242 of these (22.9%) were hospitalized. Four deaths were recorded as attributed to STEC disease: one aged 2 years and three aged more than 70 years. Three deaths were in cases recorded as having HUS, the fourth was recorded as having multiple comorbidities including COVID-19, end stage renal failure, hypertension, cardiovascular disease, but was recorded as “unknown” for HC, TTP, and HUS.

Over the 7-year period, the distribution of STEC illness between male and females was similar, with females accounting for 53% (3,068/5,769) of all cases during this time. Locality was recorded for 5,415 (94%) cases of whom 3,705 (68%) were reported to reside in urban areas.

**FIGURE 4:**
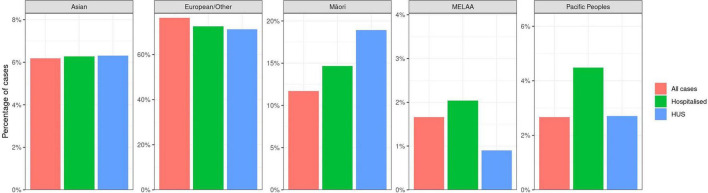
Percentages of STEC case populations represented by prioritized ethnicities Asian, European/Other, Māori, Middle Eastern/Latin American/African (MELAA), Pacific peoples by disease severity (EpiSurv extract 24th February 2023).

Figure 4 shows the relative percentages for disease presentation for each of the prioritized ethnicities. Asian and European people were similarly represented in each of the categories of all illness, hospitalization and HUS; both Pacific and MELAA people were overrepresented in hospitalization, and Māori were overrepresented in both hospitalization and HUS.

**FIGURE 5:**
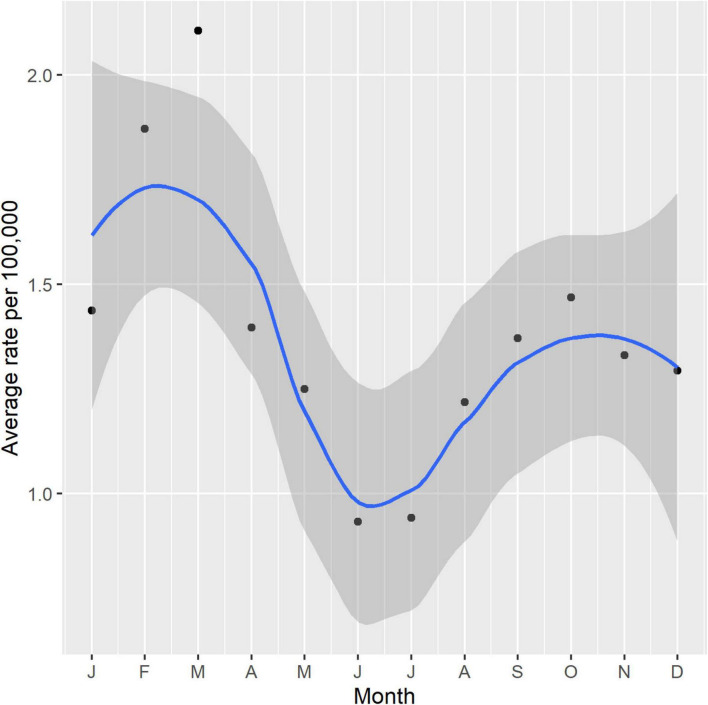
Seasonality of NZ STEC cases 2016-2022 shown as a combined average rate per 100,000 per month with 95% confidence intervals.

Figure 5 outlines the combined average rate of STEC cases per month for all cases between 2016 and 2022. A bimodal seasonality in the average rate of STEC was observed with peak notifications occurring in the NZ summer period (January-March), decreasing to the lowest rates between autumn and winter (April-September) and increasing again in spring (October-December).

### 3.2 Epidemiological typing results

#### 3.2.1 Serotyping

Prior to the introduction of WGS analysis, a full phenotypic serotype was conferred for only 75% of isolates. For the remaining 25% of isolates, phenotypic serotyping identified either an auto agglutinating rough phenotype; or no reaction with any of the antisera available and used (O, H, or both); or were non-motile and therefore could not be assigned an H type. The introduction of WGS serotype inference conferred full serotype in nearly all instances with just two ongoing non-typable outputs: O5:HNT, and ONT:H20. Investigation showed the HNT result for the O5 strains was due to these strains having an incomplete H9 *fla* gene with insufficient coverage for type calling, and the HNT designation is reported here. Investigation of the ONT:H20 showed that these findings were consistent across both SerotypeFinder and EcOH database outputs, and Nextera and Plexwell library preparations. The isolates typed as O64:H20 using in-house phenotypic methods with ST1308 being the most frequently identified ST. These are reported here as O64:H20.

During the 7-year period 2016-2022, 3,701 STEC confirmed case samples yielded 3,746 STEC strains (representing 64% of all confirmed STEC cases, 77% of all severe illness cases and 84% of all HUS cases). Of those 3,746 STEC, a total of 2,939 isolates (51% of all confirmed cases and 78% of case isolates) were analyzed by WGS.

**FIGURE 6:**
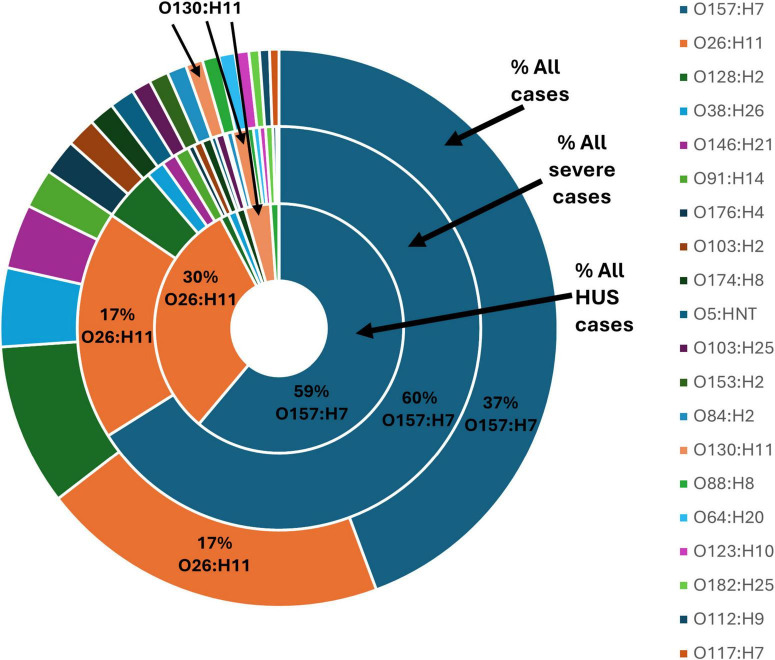
Top 20 serotypes confirmed from 3,746 STEC isolated from NZ cases of STEC infection 2016-2022, each shown as a percentage of all isolates from cases of infection, all isolates from cases of severe infection and all isolates from cases of HUS.

The serotype for all 3,746 STEC isolates was conferred either by phenotypic methods (*n* = 827) or inferred from WGS data (*n* = 2,939). A total of 148 different serotypes were observed. An additional 78 partial serotypes were conferred based on the limitations of the phenotypic method described above. Figure 6 shows the twenty STEC serotypes most frequently identified in the dataset, as well as the proportion of illness severity associated with each serotype.

STEC O157:H7 and STEC O26:H11 were the two most identified STEC serotypes representing 37% and 17% of all STEC case isolates, respectively. STEC O157:H7 predominated among the number of case isolates associated with severe illness (60%) and HUS (59%). STEC O26:H11 was the second most common serotype associated with severe illness (17%) and HUS (30%). An additional 85 serotypes were associated with severe illness and of those eight serotypes (O130:H11, 3%; O128:H2, 1%; O38:H26, 1%; O174:H8, 1%; O88:H8, 1%; O91:H21, 1%; O171:H2, 1%; O153/O178:H23, 1%) were also associated with HUS.

#### 3.2.2 Multiple STEC types within a *stx*-positive fecal sample

Identifying isolates yielding different toxin profiles within the initial ESR multiplex PCR screening led to multiple STEC types being found in case fecal samples on 44 occasions (1.2% of all *stx* + sample cultures yielding an STEC). A 45th case of multiple STEC types within a sample was identified after a public health team noted that ESR had reported O103:H2 from a sample from which the diagnostic laboratory had reported O157 encoding genes. ESR then performed serotyping on 10 *stx2* colonies from the sample and five were found to be O103:H2 and five were O157:H7. The 90 isolates from 45 cases samples were all typed, and these results are shown in Supplementary Table 1.

#### 3.2.3 *Stx* subtyping, *eae* positivity, and clinical severity

**FIGURE 7:**
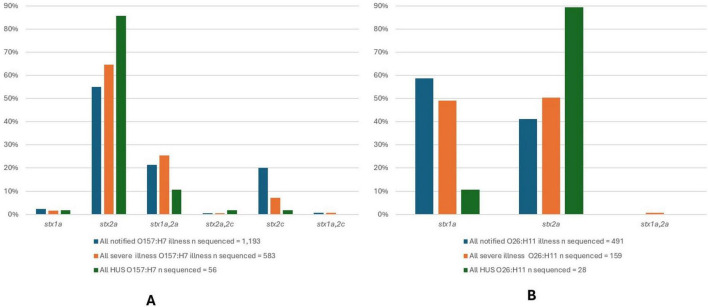
*Stx* subtypes for 1,193 STEC O157:H7 isolates **(A)** and 491 STEC O26:H11 isolates **(B)** from NZ cases of STEC infection 2016-2022, that underwent WGS analysis with each subtype shown as a percentage of all of isolates of that serotype from cases of infection, all isolates from cases of severe infection and all isolates from cases of HUS.

An overview of the percentage of STEC O157:H7 and O26:H11 isolates with different *stx* subtypes and their association with clinical disease severity is shown in Figure 7. For STEC O157:H7, the presence of *stx2a* in the absence of *stx1* predominated amongst all notified STEC O157:H7 cases (55%), as well as severe illness (65%) and HUS (86%) (Figure 7A). The presence of *stx1a* plus *stx2a* was the second most frequently observed *stx* subtype combination for STEC O157:H7: all cases (21%) and severe illness (25%) but appeared in proportionally fewer HUS case isolates than those with *stx2a* only. For STEC O26:H11, *stx1a* was observed to predominate in all STEC O26:H11 cases (59%) and but not in severe illness (50%), nor HUS (11%); and s*tx2a* predominated in HUS cases (89%) (Figure 7B).

All STEC O157:H7 and STEC O26:H11 were positive for *eae*, and together these two serotypes accounted for 89% of HUS case isolates typed by WGS.

The virulence profiles (*stx1* and/or *stx2* subtype, *eae*, and *ehx*A) for the other 18 most common serotypes identified from STEC notified cases are shown in Table 1. Twelve of these 18 serotypes had virulence profiles that were *eae* negative as did 35% (1,326/3,746) of all STEC isolated from NZ clinical cases. Six of 18 serotypes outlined in Table 1 (O103:H2, O5:HNT, O103:H25, O153:H2, O84:H2, O182:H25) had virulence profiles that included *eae*, in association with *stx1a*. None of these six serotypes were common amongst cases of severe illness and/or HUS (Figure 6).

**TABLE 1:** Toxin genes associated with 18 commonly detected NZ STEC serotypes 2016-2022.

Serotype	*stx1*	*stx2*	*eae*	*ehxA/hlyA*
O128:H2	1c/−	2b/−	−	+
O38:H26	1c/−	2b/−	−	+
O146:H21	1a/1c/−	2b/−	−	±
O91:H14	1a/−	2b/−	−	+
O176:H4	1c/−	2b/−	−	+
O103:H2	1a	-	+	+
O174:H8	1c/−	2b/−	−	+
O5:HNT*	1a	−	+	+
O103:H25	1a	−	+	+
O153:H2	1a	−	+	+
O84:H2	1a	−	+	+
O130:H11	1a/−	2a	−	+
O88:H8	1a	−	−	+
O64:H20^#^	−	2c	−	−
O123:H10	1c/−	2b/−	−	+
O182:H25	1a	−	+	+
O112:H9	1c/−	2b/−	−	+
O117:H7	1a	−	−	−

*The serotype assigned as O5:HNT (not typeable) has a partial H:9 gene by ESR pipeline analysis but coverage is insufficient for it to be called with confidence by the pipeline. ^#^The serotype referred to as O64:H20 is ONT by the ESR pipeline but ESR’s WGS analysis validation process showed this finding correlated with isolates historically typed as O64:H20 by the phenotypic typing method.

STEC O130:H11 was invariably *eae* negative and *stx2a* positive, with 30% of isolates (10/31) also carrying *stx1a*. It was the 14th most common serotype amongst all STEC cases (31 cases, 1%) but was disproportionately associated with HUS (three cases, 3%) (Figure 6). Although not amongst the twenty most common serotypes observed in the current study, STEC O91:H21 with *stx2a* but *eae* negative was seen in eight cases and was also observed to be disproportionally associated with HUS. Cases of both types occurred sporadically throughout the study period. Isolates from two cases were confirmed as being *stx2f*, an O81:H6 and an O63:H6. Both cases presented in 2016 and neither had severe illness, nor a history of overseas travel.

The composite serotype, ST, *stx* subtype, and *eae* positivity (seropathotype) of the 94 STEC isolates from 93 HUS cases are shown in Table 2.

**TABLE 2:** Composite seropathotypes results for NZ HUS cases isolates analyzed via WGS January 2016-December 2022.

Serotype	ST	*stx1*	*stx2*	*eae*	*ehxA/hlyA*	total
O157:H7	11	−	2a	+	+	47
O157:H7	13175	−	2a	+	+	1
O157:H7	11	1a	2a	+	+	5
O157:H7	10084	1a	2a	+	+	1
O157:H7	11	−	2a, 2c	+	+	1
O157:H7	11	−	2c	+	+	1
O26:H11	21	−	2a	+	+	25
O26:H11	21	1a	−	+	+	3
O130:H11	297	−	2a	−	+	2
O130:H11	297	1a	2a	−	+	1
O88:H8	446	1a	−	−	+	1
O91:H21	10469	−	2a	−	+	1
O128:H2	4748	1c	2b	−	−	1
O153/O178:H23	1642	1a	2 Not typable	−	+	1
O174:H8	10423	1c	2b	−	−	1
O171:H2	332	−	2c	−	−	1
O38:H26	10	1c	2b	−	−	1

#### 3.2.4 ST and serotype comparisons via cgMLST

WGS analysis of 2,939 STEC genomes identified 171 different ST.

**FIGURE 8:**
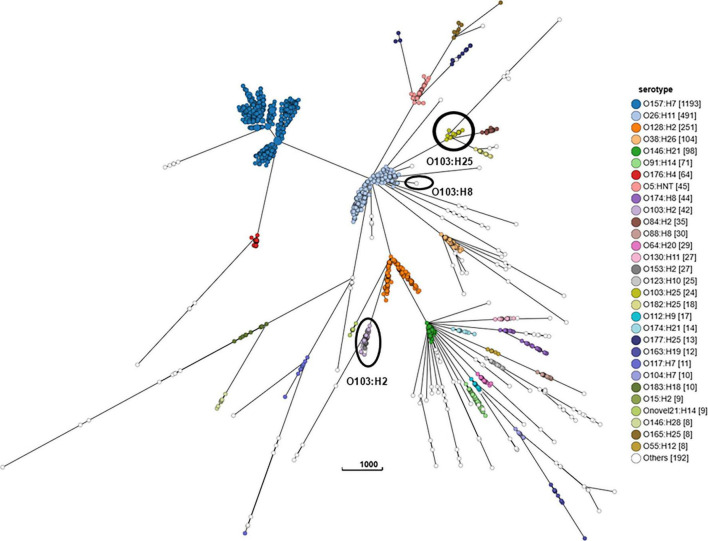
GrapeTree visualization of the cgMLST minimum spanning tree of all STEC isolated from NZ clinical cases 2016-2022, colored by serotype and highlighting the relative tree positions of O103:H25 ST343, O103:H8 ST2836, and O103:H2 ST17. The branch length scale is indicated by the number near the base of the tree.

**FIGURE 9:**
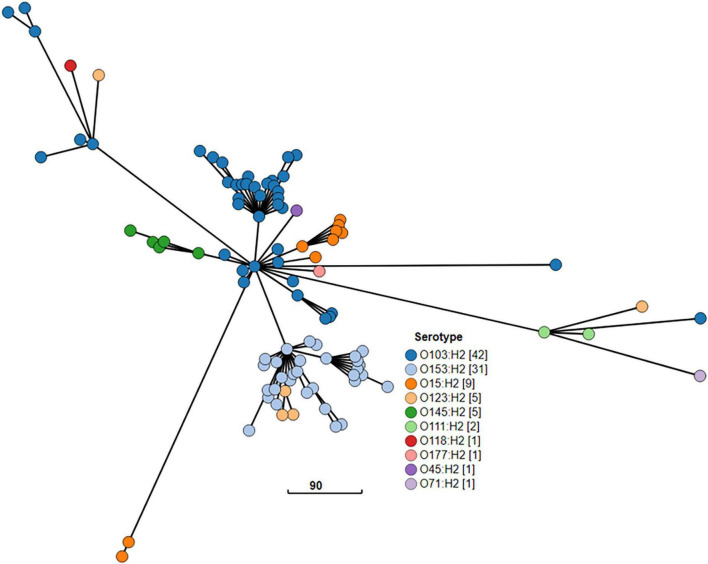
GrapeTree visualization of the cgMLST minimum spanning tree of the 10 STEC ST17 serotypes isolated from NZ clinical cases 2016-2022: O103:H2, O153:H2, O15:H2, O123:H2, O145:H2, O111:H2, O118:H2, O177:H2, O45:H2, O71:H2. The visualized branch length is calculated by the GrapeTree algorithm, and the scale is indicated by the number near the base of the tree.

**FIGURE 10:**
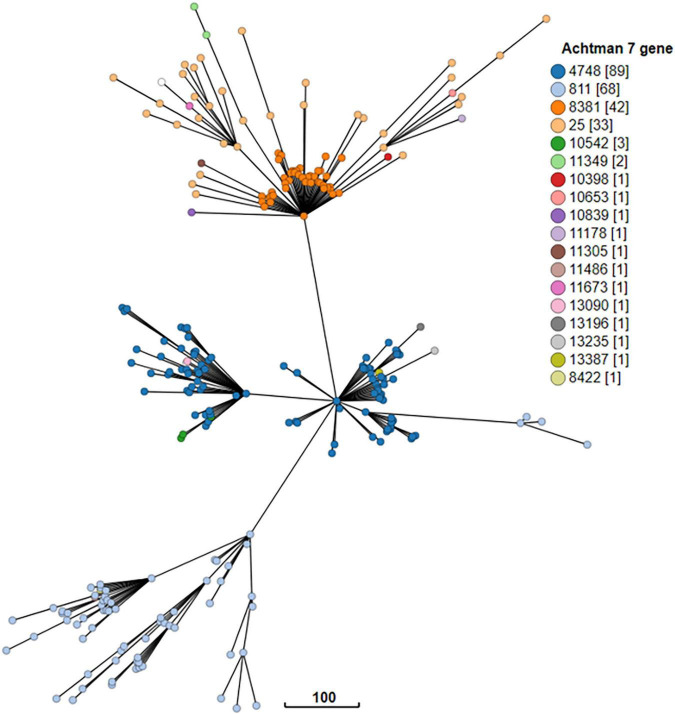
GrapeTree visualization of the cgMLST minimum spanning tree of the 18 seven gene multi locus sequence types of STEC O128:H2 strains isolated from NZ clinical cases 2016-2022. The branch length scale is indicated by the number near the base of the tree.

The cgMLST minimum spanning tree of all STEC isolated from NZ clinical cases 2016-2022 is shown in Figure 8. Three different serotypes were identified amongst the STEC serogroup O103 isolates in the dataset, each with different STs-O103:H2, ST17; O103:H8, ST2836; and O103:H25, ST343. The use of cgMLST analysis showed that the isolates within each of these O103 serotypes are genetically distinct from each other with more than 2,000 core genome differences between them (Figure 8). CgMLST analysis also showed that STEC O103:H2, ST17 formed a broad cluster with other STEC isolates of the same ST but different serotypes, all of which shared a common H type: O153:H2, O15:H2, O123:H2, O145:H2, O111:H2, O118:H2, O177:H2, O45:H2, O71:H2 (Figure 9). This clustering of isolates based on their flagella antigen suggested that ST rather than serotype is a more significant determinant of relatedness and could therefore be a primary discriminator rather than serotype. However, this was not the case with the third most detected serotype (O128:H2). As shown in Figure 10 this serotype was associated with 18 different ST that all form a broad cgMLST cluster, as the SNP variation within the housekeeping genes which drives ST variation is relatively independent of core genome relatedness. Likewise, the majority of NZ STEC O157:H7 strains are ST11 (97.7%) with the remainder being spread over 14 more recently conferred ST, and the majority of STEC O26:H11 are ST21 with < 1% spread over four recently conferred ST but these serotypes also broadly cluster by cgMLST independently of ST (data not shown).

**FIGURE 11:**
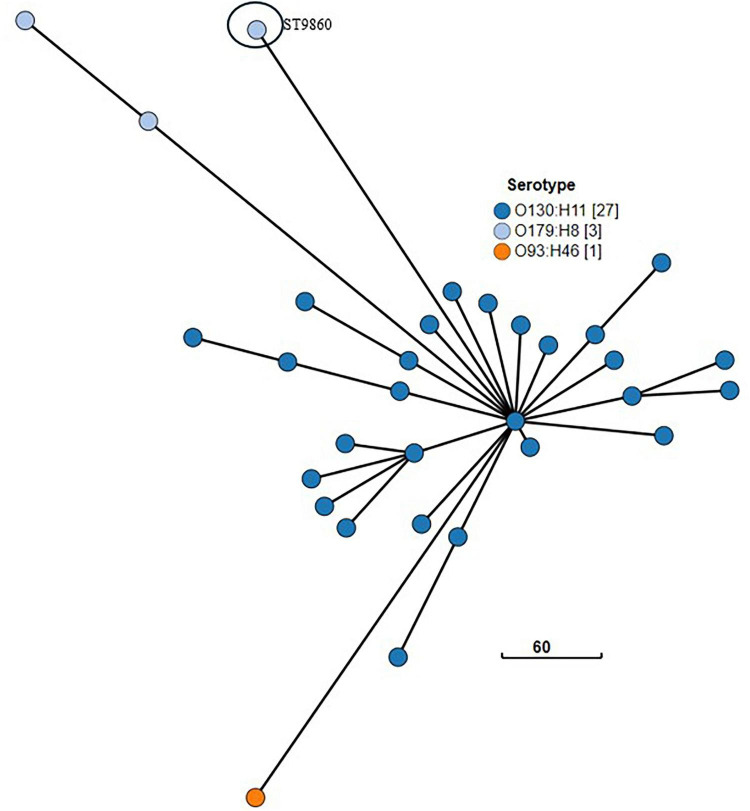
GrapeTree visualization of the cgMLST minimum spanning tree of the three serotypes of NZ clinical cases of STEC ST297 (O130:H11; O93:H46; O179:H8) and related isolate O179:H8 ST ST9860 isolated from NZ clinical cases 2016-2022. The branch length scale is indicated by the number near the base of the tree.

Some other cgMLST groups broadly cluster independently of ST and O and H type. For example, Figure 11 depicts 31 isolates which form a distinct broad cgMLST cluster, despite representing three serotypes (each a different O and H type) and two ST (O130:H11 ST297; O93:H46 ST297; O179:H8 ST297, and ST9860).

#### 3.2.5 Composite typing of ST and serotype—ST Groups

As different serotypes and/or different ST may cluster within a broad cgMLST cluster an alternative grouping based on cgMLST proximity was applied to the 2016-2022 data. This resulted in some different serotypes and STs being merged into single ST Groups, e.g., serotypes O103:H2 and O153:H2 are both in the ST17 Group. The case numbers and details of these grouping assignments are shown in Supplementary Table 2 alongside their ST Complex and EnteroBase hierarchical clustering level HC1100 cgST Complex designations in EnteroBase.

#### 3.2.6 PFGE of STEC O157:H7 isolates

Most isolates typed as either XB0079 or XB0097 (data not shown). In 2016 a cluster of STEC O157:H7 cases were linked by time and the consumption of a specific raw milk product. STEC O157:H7 was isolated from this product and that isolate and those from linked cases were all noted to be XB0079:Bl0093. No cases required hospitalization.

#### 3.2.7 STEC O157:H7 SNP analysis

SNP analysis was performed for 1,191 STEC O157:H7 confirmed case isolates and by SNP address, 399 of these (34%) were assigned to 130 5-SNP clusters comprising two or more confirmed cases from within the study timeframe. Most clusters (*n* = 79, 61%) comprised two cases, and 122 clusters (94%) comprised fewer than six cases. The PFGE outbreak cluster above was affirmed by SNP analysis as 5-SNP cluster STEC_2016_C_04 comprising six cases, and was the only cluster with an epidemiologically linked, genomically confirmed source detected during the study period. The outbreak strain was O157:H7 *stx2a*. The cluster strain has not been seen since this time and there were no other isolates within 10 SNP differences between 2016 and 2022.

Two STEC O157:H7 clusters comprised more than 10 cases: STEC_2019_C_19 (27 cases) and STEC_2020_C_30 (18 cases). STEC_2019_C_19 cases were temporally and geographically linked presenting February/March 2019 in the upper North Island. Cases ranged in age from 2 to 66 years (median 34 years); 14 cases were male (52%), and all cases resided in urban areas. Eight cases were hospitalized (30%) and none developed HUS. The cluster strain was *stx1a,2a.* Cases were interviewed using an extended trawling questionnaire, but no likely common source was identified. The strain has not been seen subsequently and there were no other isolates within 10 SNP differences between 2016 and 2022.

STEC_2020_C_30 comprised 18 cases and spanned 3 years with a single case in April 2020, five cases in February/March 2021 and 12 cases in February/March 2022. Cases were aged from 1 to 70 years (median 20 years); 10 cases (56%) were female; all cases lived in urban areas, 16 (89%) in the North Island. Nine cases (50%) were hospitalized and one case, a 9-year-old male, had HUS. No deaths were reported. The cluster strain was *stx2a.* The 2022 cases spanned six health districts and were extensively followed up by public health teams. A source was not identified, and the strain has not been seen since this time. A single 2022 isolate was within 10 SNP differences of this cluster.

**FIGURE 12:**
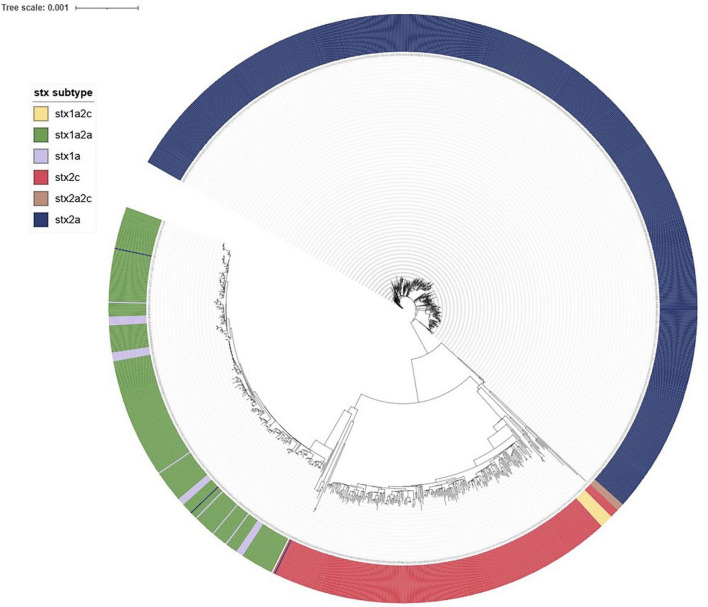
iTOL visualization of IQ-tree generated maximum likelihood tree of NZ STEC O157:H7 case strains 2016-2022 annotated to show clustering by *stx* subtype.

Figure 12 provides an overview of the population structure of STEC O157:H7 using SNP analysis. Five distinct 250-SNP clades were observed with each clade corresponding to the following *stx* subtypes: *stx2a*; *stx2a,2c; stx1a,2c*; *stx2c*; *stx1a,2a* together with *stx1a*. Three STEC O157:H7, *stx2c* isolates from cases who had recently traveled overseas were observed as genomically distinct (> 600 SNP differences from all other isolates within the dataset) and were excluded from Figure 12.

#### 3.2.8 STEC O26:H11 SNP analysis

SNP cluster analysis was performed for 488 STEC O26:H11 confirmed case isolates and 132 of these (27%) were assigned to 40 5-SNP clusters comprising two or more confirmed cases from within the study timeframe. Most clusters (*n* = 29, 73%) comprised two cases, and 37 comprised fewer than six cases. Two STEC O26:H11 clusters comprised 10 or more cases: STEC_2016_C_02 (31 cases) and STEC_2020_C_29 (10 cases).

STEC_2016_C_02 (31 cases) was not temporally clustered with cases seen throughout the study period. Most cases (*n* = 25, 81%) resided in the South Island. Cases ranged in age from 7 months to 83 years; and the median age was 5 years; 18 cases were female (58%), seven cases were hospitalized (23%), four with HUS (13%). The cluster strain was *stx2a*. Review of case report forms showed these cases to be highly rural but without an obvious linked source.

The 10 cases associated with STEC_2020_C_29 were seen between September 2020 and December 2022. Most cases (*n* = 8, 80%) resided in the South Island. Cases ranged in age from 13 months to 78 years; and the median age was 14.5 years; five cases were female (50%); four cases were hospitalized (40%), none with HUS. The cluster strain was *stx2a*. Review of case report forms showed these cases to be highly rural but without an obvious linked source.

**FIGURE 13:**
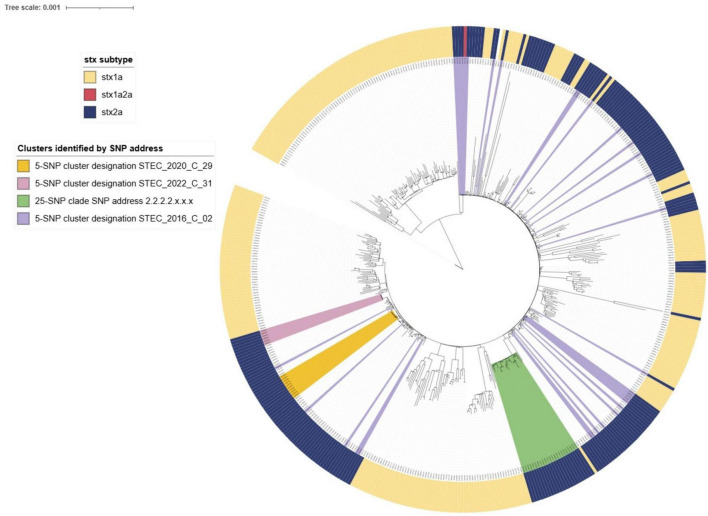
iTOL visualization of IQ-tree generated maximum likelihood tree of NZ STEC O26:H11 case strains 2016-2022 annotated to show *stx* subtype and relatedness of SnapperDB assigned clustering.

By Snapper DB SNP address, 118 of the 199 NZ derived STEC O26:H11 *stx2a* strains were within 10 SNP (SNP address 2.2.9.9.9.x.x.). Cases in this clade were predominantly from the South Island. This 10-SNP clade included STEC_2016_C_02; STEC_2020_C_29; 15 smaller clusters and 38 singletons. A further 25-SNP clade (2.2.2.2.x.x.x) comprised 23 cases, 22 of whom were North Island-based and included two small clusters and 18 singletons. When STEC O26:H11 genomic data is visualized as a maximum likelihood tree in iTOL (Figure 13) the 2.2.2.2.x.x.x clade is distinct as is the STEC_2020_C_29 cluster, and another six case cluster STEC_2022_C_31 (2.2.9.9.518.586.x); but STEC_2016_C_02 (2.2.9.9.9.9.x) is dispersed throughout the tree as are the *stx* subtypes.

The majority of the STEC O157:H7 included in SNP clustering (66%, 792/1,191) and STEC O26:H11 (73%, 356/488) isolates did not cluster within 5 SNPs of any other clinical case isolates within the study timeframe and were designated as singletons. Of the 45 isolates from cases who were recorded as having traveled overseas travel during the incubation period (10 STEC O26:H11 and 35 STEC O157:H7), 42 were singletons.

#### 3.2.9 Hybrid pathogenicity within a single STEC isolate

Using WGS analysis data, nine of the 2,939 STEC isolates were observed to possess virulence genes associated with both STEC and ETEC (Supplementary Table 3). Two isolates that were positive for *stx2e* were from travel-associated cases, whilst the remaining isolates were from cases for whom overseas travel was not indicated. Two cases (O51:H24 *stx1c,2g* and O100:H20 *stx2e*) presented with HC, whilst the remaining cases had AG only. None of the 2,939 NZ STEC isolates analyzed by WGS were observed to possess *aggR, aaiC, ipaH, bfpA*, or *lt* genes.

## 4 Discussion

### 4.1 Epidemiology

Public health surveillance of STEC illness in NZ has been impacted by methodological changes that improved the detection and characterization of a wide range of STEC in clinical cases, resulting in increasing numbers of notified STEC cases from 2015 (Figures 1, 2). By specifically targeting *stx* genes the CIDT assays progressively introduced from 2015 have an increased sensitivity over culture methods, resulting in greater numbers of non-O157 STEC detections. Concurrent to implementing CIDT, each diagnostic laboratory broadened STEC testing from a sub-group of submitted samples (based on case age and clinical information) to encompass testing all fecal samples, approximately doubling the number of samples tested for STEC (New Zealand Microbiology Network, Pers Comm). These factors have together contributed to the significant increase in non-O157 STEC cases notified between 2015 and 2022 (Figure 2). This increasing trend of STEC detection, particularly for non-O157 STEC, associated with the implementation of CIDT has also been reported in other countries including: United States (Iwamoto et al., 2015; Shah et al., 2024), Ireland (Rice et al., 2016), England (Vishram et al., 2021; King et al., 2025), Denmark (Kjelsø et al., 2024). A statistical evaluation of NZ STEC notification data for the period 2015-2020, confirmed that the increase in detections (and subsequent notifications) observed since 2015 was due to diagnosing more people with STEC infection (many of whom would not previously have been diagnosed), rather than an underlying increase in STEC infections in NZ. In addition, areas that had not transitioned to CIDT nor increased the sample population screened for STEC, showed no increase in notification rates (Horn et al., 2021).

Following the increased incidence of STEC from 2015 to 2019, there was a marked decrease in incidence in 2020 (16.6/100,000), which coincided with NZ implementing national protection measures in response to the COVID-19 pandemic in March 2020 (Jefferies et al., 2020). During this time, diagnostic laboratories’ staff and resources were prioritized to establishing and performing testing for COVID-19, and other testing was deprioritized. This, coupled with people not being able to readily access face-to-face medical assistance, meant that for a period of 2 months, there was a reduction in testing cases of acute gastroenteritis and a concomitant decrease in STEC case notifications and enteric pathogen isolate referral to ESR (Figure 2).

Once NZ began de-escalating the national restrictions from 13 May 2020 (Jefferies et al., 2020) enteric testing and isolate referral gradually recommenced with STEC notifications increasing nearer to the levels seen pre-COVID-19. However, Government behavior guidance at varying levels continued through to the end of 2021 with restrictions such as social distancing impacting on people’s frequency of exposure to STEC by reducing person-to-person transmission, other than within households, and reducing urban dwellers access to rural type exposures. The dynamics of foodborne transmission would also have been impacted as access to restaurants and takeaway outlets were (at times) limited resulting in more meals prepared and eaten at home. These factors would all have contributed to the lower rate of STEC notified cases observed throughout the remainder of 2020 and 2021 compared with 2019. Similar downward trends were observed in other countries during the COVID-19 pandemic including France (Cohen et al., 2022), Japan, (Higurashi et al., 2023), United States (Ray et al., 2021), Canada (Dougherty et al., 2023), Germany (Ullrich et al., 2021) and others summarized in a 2024 review (Lazarakou et al., 2024).

In addition, NZ border entry was restricted from March 2020 to mid-2022 accounting for the decrease in cases of overseas-acquired STEC infections recorded in the latter 3 years of the study data (2.6% of cases compared with the pre-pandemic study period of 2016-2019, where 11.9% had a recent overseas travel history). Apparent in the travel data results is that some fields of the case report form were insufficiently completed during the pandemic years, and this was most likely due to two reasons: public health teams were more focused on pandemic activities; and overseas travel was deemed a minimal risk as border entry was restricted.

NZ’s 2022 incidence of STEC infection of 19.9 cases per 100,000 population is substantially higher than rates reported for many other industrialized regions including Australia (3.2 per 100,000 population; 2022) (Shrestha et al., 2024), the United States of America (6.3 per 100,000 population; 2019) (Tack et al., 2020) and Europe (overall European Union/European Economic Area countries (*n* = 29) was 2.5 cases per 100,000 population); but two European countries have similarly high rates Denmark (22.6 per 100,000 population) and Ireland (17.6 cases per 100,000 population) (European Centre for Disease Prevention and Control, 2024). However, caution must be taken when comparing data from other countries as notification systems, case definitions and testing regimes may differ.

All observed trends in changes in STEC notification rates between 2015 and 2022 must be considered in the context of changes to testing approaches described above. However, what can be concluded is that the current infection rate reflects better case finding methods rather than an emergent source; STEC causes a high rate of illness in NZ; and the majority of NZ STEC illness is locally acquired.

Despite NZ diagnostic laboratories broadening the scope and sensitivity of fecal tests screened for STEC when introducing CIDT, there are variations between laboratories in the level of diagnostic stewardship applied to fecal samples, with some processing all samples that are unformed, and others only processing samples if one of a range of criteria is met. These include hospitalization, HUS, a history of diarrhea for over 1 week, bloody stools, underlying immunocompromising condition, high-risk settings or person (e.g., food handler, childcare, rural, overseas travel, pregnancy, specific public health request, aged under five or over 65 years) (New Zealand Microbiology Network, pers comm). As a result, NZ notified case data may under-represent the true burden of STEC infections. A NZ study estimated that for each diagnosed case of infectious AG there are up to 100 untested infections (Adlam et al., 2011). Despite these inconsistencies, the NZ model of STEC case-finding based on direct laboratory notification of cases with samples positive for *stx* genes enabling local public health teams to follow-up in real-time and determine case classification based on clinical presentation, (i.e., case/not a case) is a relatively effective and efficient method.

STEC is a burden for both personal and public health in NZ. Severe illness (HC, and/or TTP and/or HUS) was reported for 25% of all cases and 23% of cases were hospitalized. The greatest level of morbidity was observed in those less than 5 years old (Figure 3). However, all age groups were impacted (median age for AG and severe illness being 33 and 28 years respectively), and mortality was more associated with those more than 70 years, with three of the four recorded deaths, over the study period in this age group. One of those who died was not reported to have HUS but was reported to have several co-morbidities. As co-morbidity information is not actively collected as a part of STEC surveillance activities in NZ, this finding cannot be further contextualized.

HUS was reported in 1.9% of all NZ STEC cases (*n* = 111, median age 3 years). This seemingly low percentage is likely due to the NZ denominator being broader than other countries’ as it includes all symptomatic cases of all STEC types, diagnosed predominantly by CIDT (*n* = 5,769). This presumption is strengthened by our finding of an NZ annualized HUS incidence rate of 2.86/100,000 in the under 5 years age group for the years 2016-2022. A rate which concurs with that previously reported by the New Zealand Pediatric Surveillance Unit which undertook active surveillance of childhood HUS from January 1, 1998 to December 31, 2020. This team reported a mean incidence rate of STEC HUS in NZ children less than 5 years of 2.85/100,000 population for the period 2009-2020 and noted this rate to be higher than that reported from other Western countries (Wong, 2022; Wong et al., 2023). They also reported that pediatric STEC HUS is an important cause of acute kidney injury in NZ with 56% of cases requiring dialysis and some of these progressing to chronic kidney disease leading to long term personal health issues and a concomitant ongoing burden on the health system.

Prioritized ethnicity differences in the percentages of STEC hospitalization and HUS are evident in Figure 4. Those cases identifying as Māori, Pacific People, and MELAA have a higher proportion of hospitalizations compared with other prioritized ethnicities. Our data indicate the percentage of STEC illness in Māori is disproportionately low (11.2%) compared with the percentage population identifying as Māori across the 7 years of the study period (16.9%) (Statistics New Zealand, 2024) and Māori are overrepresented in HUS cases (18.9%). Disparities in access to primary healthcare providers for Māori are well documented (Ellison-Loschmann and Pearce, 2006; Baker et al., 2012; Jeffreys et al., 2021; Health New Zealand Te Whatu Ora, 2024), and may explain the lower percentage of STEC AG detections for Māori. A 2021 report identified multiple barriers to primary healthcare for both Māori and Pacific Peoples and resultant higher risk of hospitalization for both ethnicities. MELAA were not included in this evaluation (Jeffreys et al., 2021). The higher percentage of HUS in the Māori population requires further investigation.

NZ STEC infection shows bimodal seasonality (Figure 5) with the lesser peak being during the spring, possibly associated with ruminant exposure during the calving/lambing season, but the greater peak is during the summer months which is more supportive of environmental (outdoor recreational activity) or food exposures (barbecues, salads etc.) Further work is required to explore STEC-type-specific seasonality. Rural-dwelling New Zealanders experience STEC infection disproportionate to the percentage population living in urban regions. NZ data show that 16% of New Zealanders reside at rural addresses (Environmental Health Intelligence New Zealand, 2024) and our data show that 32% of STEC cases reside rurally. This is not unexpected given the primary reservoir is ruminant livestock (Griffin and Tauxe, 1991; La Ragione et al., 2009) and previous NZ studies, including a case-control study have reported that rural activities including contact with cattle feces and living near cattle are significant risk factors for STEC infection (Jaros et al., 2013; Richardson et al., 2016). Multiple international studies have also reported geographical and/or seasonal differences with STEC infection rates, particularly with rural risk factors (Byrne et al., 2014; Elson et al., 2020; Cleary et al., 2021). Ruminant dense environments increase the risk of STEC infection independent of ruminant contact (Ward et al., 2025). In the South Island, dairy cattle numbers increased by over 90% between 2002 and 2018 from 1.3 to 2.6 million with some areas now showing high cattle density (Environmental Health Indicators New Zealand, 2019; Livestock Improvement Corporation Limited, and DairyNZ Limited, 2022). A 23-year study of pediatric STEC HUS in NZ found the greatest risk for those STEC infections came from rural exposure and that increasing HUS numbers in areas of the South Island mirrored increasing intensification of dairying in those areas (Wong et al., 2023).

### 4.2 Microbiology and genomics

WGS analysis for STEC at ESR has proven to be a cost-effective replacement to phenotypic serotyping which was inefficient and expensive due to the high cost of purchasing and shipping the range of O and H antisera required to perform full serotyping to the reference laboratory; the time and personnel input required to perform full serotyping were substantive; the outputs were poor (25% of results were only partial due to either autoagglutination, or absence of positive results); and the typing data provided only low level discrimination. For a smaller expenditure and within a single process (which by doing two mixed organism NextSeq runs per week is faster than phenotypic serotyping), we attain full serotype; additional virulence information such as *stx* subtype and other *E. coli* virulence genes; genomic grouping in the form of ST, and broad and fine clustering based on the core genome; and other data yet to be fully analyzed such as antimicrobial resistance genes, other virulence genes, and plasmid information. From this rich genomic dataset, we have been able to perform the detailed characterization of 2,939 STEC from clinical cases presented here.

Our WGS data show that serogroup (O antigen only) is a poor discriminator within STEC as some serotypes, including two common NZ serotypes—O103:H2 and O153:H2 (both ST17)—are more closely related than serotypes within the same O group but with different H antigens—for example O103:H2 (ST17) and O103:H25 (ST343). This supports the European Food Safety Authority’s (EFSA) conclusion that serogroup in isolation should not be used as an epidemiological typing marker (EFSA et al., 2020).

A typing scheme for epidemiological purposes must have sufficient discriminatory power to group isolates most likely associated with a common source while excluding those that are not. Serotyping is a useful step on that journey. ST is also a useful step, but Figures 8-11 demonstrate the importance of assessing the relatedness of isolates based on cgMLST similarity in conjunction with both serotype and ST for epidemiological and surveillance purposes. We have shown that many serotypes may broadly cluster within a single ST (such as ST17)—indicative of recombination activity within the O-Antigen biosynthesis gene cluster in otherwise closely related STEC isolates. This was first reported in STEC isolates from German cattle (Geue et al., 2017) and more recently in emerging serotype variants of the highly virulent German O104:H4 outbreak strain (Lang et al., 2023). Conversely our data show that some serotypes comprise genomically distinct strains (Supplementary Table 2: O163:19, O117:H7). In other situations (such as in our O128:H2 dataset), a change in ST may be due to a single nucleotide variation in one of the house-keeping genes used in the seven gene MLST scheme with no major concomitant shift in the core genome structure.

These findings demonstrate that neither serotype nor ST should be used to define STEC strains for the purposes of epidemiological investigation. Instead, we propose that “ST Group” (Supplementary Table 2) based on core genome relatedness for serotype/ST combinations be used as a defining, discriminatory step from which finer typing techniques such as SNP analysis can be leveraged; and that seropathotype (ST Group plus *stx* subtype) be considered when considering clinical severity. ST Complex designation is used here for comparative purposes in preference to clonal complexes (CC) which were generically described (Feil et al., 2004) prior to the introduction of the widely accepted *E. coli* seven gene MLST scheme, which also introduced the ST Complex as the next tier in genomic grouping within this species (Wirth et al., 2006).

As shown in Supplementary Table 2 some of our ST Groups correlate with the ST Complex recorded in EnteroBase (Zhou et al., 2020), but not all the serotypes/STs seen in our dataset have been assigned to an ST Complex. The EnteroBase hierarchical clustering level HC1100 cgST Complex results, based on cgMLST (Achtman et al., 2022), correlate with our ST Grouping assignment; however, EnteroBase does not readily link different STs that share a common HC1100 cgST Complex. For example, ST297 and ST9860 are both designated HC1100 cgST Complex 1081 and having recognized their relatedness we have grouped them together as the ST297 group.

Our data reinforce that it is important for each country/region to identify its local common strains as the NZ “Top 20” serotypes (Figure 6) and ST Groups (Supplementary Table 2) are distinct from those reported for other areas (CDC, 2024; European Centre for Disease Prevention and Control, 2024). An early example of this was the preliminary typing of STEC O84 using culture-based methods coupled with plasmid mapping which indicated the presence of an NZ-specific clone of the *stx1a* STEC O84:H2 seropathotype (Cookson et al., 2006a).

The dominant NZ serotypes (O157:H7 and O26:H11) are invariably positive for *eae* (the gene encoding for intimin which is associated with heightened virulence in STEC); and *stx2a* strains are more associated with severe illness. STEC O157:H7 and STEC O26:H11 which are not subtype *stx2a*, are less associated with severe presentations (Figure 7) and appear to have the same clinical impact as other *stx2a* negative, *eae* positive STEC (Figure 6 and Tables 1, 2).

Other serotypes in our dataset which are invariably *eae* negative, but which are *stx2a* such as O130:H11 are also more likely to be associated with severe illness than *stx1a*, *eae* + positive STEC such as O103:H2 and O103:H25 (Figure 6 and Table 1). Our data show that common *eae* negative serotypes that are associated with *stx1c* and/or *sx2b* are disproportionately less likely to be associated with severe illness (Tables 1, 2 and Figure 6). Our data also show that severe illness is not exclusively seen in association with *stx2a* positive strains. However, our incidental finding of 45 cases who were excreting multiple STEC seropathotypes does not preclude the possibility that cases of severe disease from whom strains with *stx* subtypes other than *stx2a* or *stx2d* are cultured are also carrying additional STEC undetected by the current process. Our data support the 2020 conclusions of EFSA—that serogroup (O type) cannot be used as a predictor of clinical outcome; the presence of the intimin gene (*eae*) is not essential for severe illness (defined as bloody diarrhea (BD), hemolytic uremic syndrome (HUS) and/or hospitalization); and that isolates positive for any *stx* gene subtypes may be associated with severe illness (EFSA et al., 2020). A limitation of our study is that our data do not include co-infection information, thus these STEC cases may also have been positive for other enteric pathogens which could have contributed to their clinical presentation. In addition, the genomes were not evaluated for the presence and potential impact of multiple copies of stx genes (Ashton et al., 2015).

STEC seropathotype O26:H11 ST21 *stx2a* emerged in NZ during this 7 year study period (Wright et al., 2021; Wright et al., 2022) and is now associated with more cases of severe STEC disease in NZ than STEC O26:H11 *stx1a* (Figure 7B). The emergence of “hypervirulent” O26:H11 *stx2a* worldwide was highlighted in 2013 (Bielaszewska et al., 2013) and it was originally noted that these international strains were associated with ST29 and ST21 in similar numbers. More recently a predominance of O26:H11 ST21 *stx2a* in England has been reported (Rodwell et al., 2023) The only STEC ST29 in our dataset was an O123:H11 *stx1a*; but non-toxigenic NZ O26 ST29 strains have been reported from bovine samples (Browne et al., 2019).

Hybrid pathogenicity is an occasional finding in NZ and its presence has not resulted in disproportionately severe illness. Eight hybrid toxicity strains were identified, and all were uncommon serotypes. Two of the isolates were *stx2e* positive, the variant normally associated with pig edema disease (Casanova et al., 2018), both cases had a recent history of overseas travel. One of the hybrid serotypes identified, O15:H16, correlated with a previous hybrid STEC report (Nyholm et al., 2015); two of the eight isolates, O187:H28 ST200 *stx2g*, and O15:H16 ST325 *stx2g*, correlated with serotype, *stx* and heat stable toxin genes described in a more recent report (Bai et al., 2019); and four of the eight correlated with hybrid strains described in a 2025 publication (Rodwell et al., 2025).

We acknowledge the international significance of mucus/elastase activatable *stx2d* (Feng et al., 2017; Sánchez et al., 2017) in severe clinical outcomes—also in the absence of *eae* (Feng et al., 2017). This *stx2* subtype is comparatively rare in NZ and is yet to be associated with severe illness, but we remain vigilant of its potential.

Our results suggest *stx2f* is rare in NZ and historical data (not shown) appear to confirm this. However, the reference laboratory only screens isolates that are *eae* positive, so *eae* negative *stx2f* isolates will not have been detected. No *stx2f* STEC have been detected in NZ since 2016, however this may not be a genuine representation of prevalence. It may instead be an artifact brought about by the progressive implementation of CIDT, as it would appear that commercial panels in use do not contain an *stx2f* target (Cointe et al., 2020; Ausdiagnostics, 2023; BioMérieux, 2024). It is acknowledged that *stx2f* strains may cause severe illness (Friesema et al., 2015; Den Ouden et al., 2023) and that NZ will need to be vigilant to the possibility of *stx2f* strains in HUS cases that are *stx* gene negative at the diagnostic laboratories.

Fine clustering via SNP analysis has become a routine part of STEC O157:H7 and O26:H21 genomic analysis. Whilst 66% STEC O157:H7 and 73% STEC O26:H11 case isolates were singletons (more than five SNP differences from any other isolate), SNP analysis proved successful in demonstrating genomically related strains with a recent common ancestor that could be due to a common source. Investigations associated with the four large (≥ 10 cases) clusters showed very different demographics between the two serotypes. Both STEC O157:H7 clusters were predominantly urban and from the North Island and had median ages of 20 and 34 years. Both STEC O26:H11 clusters were predominantly rural South Island locations and had median ages of 4 and 6 years. These differences highlight the importance of evaluating the epidemiological context of different STEC types separately rather than as a generic group. In addition, the cgMLST tree of all STEC shown in Figure 8 demonstrates that O26:H11 and O157:H7 are genomically remote from each other and Figures 12, 13 highlight that each has different phylogeny with STEC O157:H7 strains clearly clustering by *stx* subtype suggesting each subtype evolved independently. NZ STEC O26:H11 strains are not clearly differentiable by *stx* subtype mirroring previous reported diversity within ST21 clades particularly within ST21C1 due to repeated *stx2* acquisition in multiple lineages (Ogura et al., 2017; Long et al., 2022). It is anticipated that future work will be undertaken to compare the genomes of NZ and international O26:H11 ST21 *stx2a* strains to determine the relatedness of NZ endemic strains to those found elsewhere. It is apparent that STEC are a heterogeneous group of organisms and similarities between types cannot be assumed. Likewise, not all typing methods may be appropriate for all ST Groups and the disparities between Snapper DB and IQ-Tree cluster determination in O26:H11 strains apparent in Figure 13 require further investigation. This will include reviewing the impact of using a reference genome prepared from a local STEC O26:H11 strain on the clustering outputs.

For other serotypes, cgMLST can be used for fine clustering progressing to SNP analysis if indicated by genomic or epidemiological information. However, to achieve a robust and epidemiologically useful outcome, SNP-based clustering requires a reference strain which has a similar core genome to the test population. Our data suggests that ST Group (based on cgMLST similarity) rather than serotype is a useful determinant for deciding which organisms from a broad STEC population and can be combined into the one reference-based SNP analysis.

### 4.3 Limitations of STEC culture methods and findings of multiple seropathotypes within a stx positive fecal sample

The current method of using two selective media and testing 12 colonies per sample for *stx* genes (Rivas et al., 2024) yields a culture positivity rate of 64% of all CIDT positive samples. Reasons for not isolating a greater percentage of STEC include: (1) the number of live STEC organisms in the sample may be insufficient to detect by the culture method as CIDT methods amplify the *stx* gene irrespective of whether the source organism is healthy, sub-lethally injured, or dead; (2) the inhibitors in the chosen selective culture media may suppress some STEC, allowing generic *stx*-negative *E. coli* to overgrow and obscure these.

It is acknowledged that using additional culture media (including enrichment broths, and less selective culture plates) coupled with increasing the number of colonies tested could improve the organism recovery rate. However, the current practice is a compromise based on financial ability, and it is cost prohibitive to routinely do more at the present time.

Multiple seropathotypes were found in cases’ fecal samples on 45 occasions. This finding was based on screening 12 isolates per sample for *stx1*, *stx2*, and *eae* and further identifying those with different toxin profiles. This crude screening is likely a gross underrepresentation of the actual number of cases with multiple STEC strains as multiple strains were not sought if all isolates tested had the same toxin profile. We may therefore be selecting *stx2b* strains for typing when a *stx2a* strain may also be present. The sole exception is case 37, Supplementary Table 1, where ESR reported O103:H2 but the primary laboratory had found O157 encoding genes. Ten *stx2* colonies from the sample were then serotyped and five were found to be O103:H2 and five were O157:H7.

Future research indicated by these findings include: (1) developing a sensitive, rapid and low cost *stx2a* test which could be applied at the clinical laboratory on all *stx* gene positive fecal samples to expedite clinical support and public health follow up of cases more at risk of severe illness; (2) determining the individual significance of each type when two STEC strains are found in the same sample; (3) determining whether when a *stx1c* and/or *stx2b* strain is the only finding in a case of severe illness there is actually a second more acknowledged virulent strain present that was initially overlooked by the selection method; (4) evaluating if some combinations of STEC strains together enhance or reduce the virulence of each in isolation. Work is planned to evaluate the relative virulence of various seropathotypes using 3D microfluidic cell models (Leung et al., 2022).

### 4.4 Local knowledge and data gaps

The data presented here demonstrates that we now have a detailed knowledge of the STEC strains associated with clinical disease in NZ and a more superficial knowledge of the cases themselves. The purpose of the case report form (ESR, 2023) is to gain high level information for surveillance purposes. It is not designed for detailed, research level source attribution. The use of detailed trawling questionnaires can be activated if a surge in cases is noted, or genomics indicate fine clustering of case isolates. In this situation the delay between symptom onset and questionnaire implementation can lead to problems in memory recall. In addition, as potential risk activities may be common across the NZ population, a control group is important for gaining accurate and specific risk information for source attribution. A case control study where detailed interviews are undertaken sooner, and cases are matched to asymptomatic controls would address these issues. Such a study was completed in NZ in 2012 and 100 of the 113 eligible cases (123 STEC cases were notified that year) were infected with STEC O157:H7 (88% of the cases); statistically significant animal and environmental risk factors were identified including exposure to cattle, contact with animal manure and contact with recreational waters, however, food associated risk factors including eating meat, were found to be insignificant (Jaros et al., 2013). At that time, diagnostic testing of clinical samples for STEC was usually performed for only a selection of AG cases that met age and clinical presentation criteria and was done using culture-based methods that primarily targeted STEC O157:H7. Therefore, those findings may be of little relevance to the NZ STEC landscape of today as case numbers, causative types and our environment have changed; and investigative technologies (both diagnostic and typing) have vastly improved in the intervening years. A new NZ case-control study, covering both a broad geographical area and population representation, and all seasons, is advised. Such studies have been recently reported from England (Kintz et al., 2023) and Denmark (Kjelsø et al., 2024), but their relevance to the NZ situation cannot be assumed.

Few large clusters were noted indicating that STEC infection in NZ is predominantly sporadic. But the detailed epidemiology of NZ STEC infection is currently unclear, particularly pathways to acquisition and their relative importance. Estimating the proportion of STEC cases attributed to food sources and transmission pathways in NZ using expert elicitation resulted in estimates ranging from 5% to over 60%, with most participants suggesting that food pathways are more likely to contribute to non-O157 STEC infections than to STEC O157 infections. Based on this colloquium discussion the current percentage attribution to food sources used in NZ is 20% STEC O157:H7, and 40% other STEC (Soboleva, 2021).

On only one occasion was an epidemiologically linked source genomically confirmed (raw milk). This is in part due to the dearth of potential source information and genomes available for comparison. The strength of applying STEC WGS in food safety and public health surveillance is in the efficiency of WGS in detecting genomic relationships; but its ability to support epidemiology is dependent on the representativeness of the comparative non-human dataset (Nouws et al., 2023). The current NZ non-human comparative genomic dataset set is small and not representative as there is currently no national STEC food monitoring program in NZ. Recent NZ meat and environment studies have focused on the USDA Top 7 STEC serogroups with specific attention to STEC O157:H7 and STEC O26:H11 (Irshad et al., 2012; Irshad et al., 2014; Jaros et al., 2014; Jaros et al., 2015; Irshad et al., 2017; Browne et al., 2018; Jaros et al., 2018; Browne et al., 2019; Browne et al., 2021) and little comparative genomic analysis has been undertaken between those isolates and recent human case isolates. Genomic comparisons done on a limited number of bovine raw meat isolates show that some human and bovine strains of STEC O157:H7 are genomically very similar and share a recent common ancestor (Wright et al., 2024) which is unsurprising given it has been recognized from the 1980s that bovines and other smaller ruminants are the primary reservoir for STEC (Griffin and Tauxe, 1991; La Ragione et al., 2009). Without epidemiological support, such a finding does not indicate causation. No STEC were reported from three NZ studies of leafy green produce, but each was of limited scope (McIntyre and Cornelius, 2009; D’Sa et al., 2015; Hewitt et al., 2015).

The need to consider animals and environs beyond the home/farm such as wildlife and aquatic sources as STEC reservoirs has been highlighted (Kim et al., 2020) and some NZ work has been done in this space. *Stx* genes have been reported in samples from flies and bird droppings (Rapp et al., 2021); river waters from around NZ (Leonard et al., 2020); and other various freshwater sites (Cookson et al., 2022) (Cookson et al., 2024). These recent STEC findings, coupled with the findings of many STEC serotypes in historical NZ studies of point-of-sale meat, and environmental samples (Brooks et al., 2001; Bennett and Bettelheim, 2002; Cookson et al., 2006b; Rivas et al., 2014) support the importance of undertaking more food and environmental sampling. Such sampling must culminate in STEC isolation followed by WGS to provide the fine typing information essential for comparison with clinical isolates to resolve sources and pathways to STEC infection in NZ.

## 5 Conclusion

STEC infection is a significant endemic disease in New Zealand.

By combining existing NZ case epidemiological data with isolate typing data, particularly that derived from WGS, we have demonstrated the importance of shifting the focus from a select number of STEC serogroups to composite seropathotypes—based on full serotype, ST, cgMLST relatedness and *stx* sub-toxin profile—to assist in understanding both the role of each type in disease severity, and relationships across historical type groups.

Doing so enables a better understanding of the types of STEC associated with disease and the relative significance of each to causing severe illness. This knowledge could be used to assist in future prioritizing of clinical and public health resources and to create targeted, rather than blanket, exclusion criteria.

Whilst our current work has demonstrated a new depth of knowledge on the STEC seropathotypes associated with STEC clinical cases of varying severity in NZ, it has also highlighted the paucity of comparator strain information from our food and environment.

Interrupting the pathways from reservoir to humans is key to reducing the burden of STEC disease. Further food and environmental STEC testing and strain comparison with case isolate genomes coupled with an updated case control study should elucidate transmission pathways to infection from the known ruminant reservoirs and inform the interventions needed to reduce STEC disease in NZ.

## Data Availability

Reads from isolates sequenced as part of this study have been submitted to the National Center for Biotechnology Information (NCBI) under BioProject PRJNA1068194.
