# Supplementary material for: Shiga toxin-producing Escherichia coli illness in Aotearoa | New Zealand, 2016-2022: epidemiological, genomic and traditional typing analyses provide insight into a significant endemic disease while highlighting knowledge gaps
Source: Front Microbiol. 2025 Jul 2;16:1605469. doi: 10.3389/fmicb.2025.1605469 (PMC12263908; doi:10.3389/fmicb.2025.1605469)
Supplement: Supplementary file 1 [file Data_Sheet_1.docx]

**Shiga toxin-producing *Escherichia coli* illness in Aotearoa | New Zealand, 2016-2022: Epidemiological, genomic and traditional typing analyses provide insight into a significant endemic disease while highlighting knowledge gaps.**

**Authors: Jacqueline (Jackie) Wright; David Duncan; Hugo Strydom; Shevaun Paine; Sarah Jefferies; Joep de Ligt; Lucia Rivas; Michael Addidle; Adrian L. Cookson; David Winter; Hilary Miller; Geraldine Casey; Jing Wang**.

Supplementary Table 1. NZ STEC cases identified as having multiple STEC types 2016-2022.

| Case | Serotype* | Seven gene MLST* | *stx1** | *stx2** | *eae* | *ehxA/hly*A | Heat stable toxin (*st*) |
| --- | --- | --- | --- | --- | --- | --- | --- |
| 1 | O38:H26 | 10 | 1c | - | - | + | - |
| 1 | O174:H8 | 10423 | 1c | 2b | - | - | - |
| 2 | O91:H14 | 33 | 1a | 2b | - | - | - |
| 2 | O88:H8 | 446 | 1a | - | - | + | - |
| 3 | O38:H26 | 10 | 1c | - | - | + | - |
| 3 | O38:H26 | 10 | 1c | 2b | - | + | - |
| 4 HUS | O157:H7 | 11 | - | 2a | + | + | - |
| 4 HUS | O26:H11 | 21 | 1a | - | + | + | - |
| 5 | O174:H8 | 13 | 1c | - | - | + | - |
| 5 | O26:H11 | 21 | - | 2a | + | + | - |
| 6 | O157:H7 | 11 | - | 2c | + | + | - |
| 6 | O103:H2 | 17 | 1a | - | + | + | - |
| 7 | O26:H11 | 21 | - | 2a | + | + | - |
| 7 | O182:H25 | 300 | 1a | - | + | + | - |
| 8 | O5:HNT | 342 | 1a | - | + | + | - |
| 8 | O64:H20 | 1308 | - | 2c | - | - | - |
| 9 | O91:H14 | 33 | - | 2b | - | + | - |
| 9 | O130:H11 | 297 | 1a | 2a | - | + | - |
| 10 | O182:H25 | 300 | 1a | - | + | + | - |
| 10 | O112:H19 | 5296 | - | 2d | - | - | - |
| 11 | O91:H14 | 33 | - | 2b | - | + | - |
| 11 | O91:H14 | 33 | 1a | 2b | - | + | - |
| 12 | O128:H2 | 811 | 1c | 2b | - | - | - |
| 12 | O146:H21 | 829 | 1c | - | - | - | - |
| 13 | O128:H2 | 8381 | 1c | 2b | - | + | - |
| 13 | O128:H2 | 13235 | 1c | - | - | - | - |
| 14 | O176:H4 | 57 | 1c | - | - | + | - |
| 14 | Onovel21:H14 | 7010 | - | 2b | - | - | - |
| 15 | O128:H2 | 8381 | 1c | 2b | - | + | - |
| 15 | O146:H21 | 10438 | 1c | 2a | - | - | - |
| 16 | O157:H7 | 11 | 1a | 2a | + | + | - |
| 16 | O146:H21 | 829 | - | 2a | - | + | - |
| 17 | O38:H26 | 10 | 1c | 2b | - | + | - |
| 17 | O26:H11 | 21 | - | 2a | + | + | - |
| 18 | O26:H11 | 21 | - | 2a | + | + | - |
| 18 | O5:HNT | 342 | 1a | - | + | + | - |
| 19 | O157:H7 | 11 | - | 2a | + | + | - |
| 19 | O130:H11 | 297 | - | 2a | - | + | - |
| 20 | O157:H7 | 11 | - | 2a | + | + | - |
| 20 | O146:H21 | 12507 | - | 2b | - | - | - |
| 21 | O38:H26 | 10 | 1c | 2b | - | + | - |
| 21 | O91:H14 | 33 | - | 2b | - | - | - |
| 22 | O103:H2 | 17 | 1a | - | + | + | - |
| 22 | O8:H9 | 23 | - | 2e | - | - | - |
| 23 | O153:H2 | 17 | 1a | - | + | + | - |
| 23 | O174:H21 | 6639 | - | 2a | - | - | - |
| 24 | O88:H8 | 446 | 1a | - | - | - | - |
| 24 | O128:H2 | 811 | 1c | 2b | - | + | - |
| 25 | O157:H7 | 11 | - | 2c | + | + | - |
| 25 | O111:H2 | 17 | 1a | - | + | + | - |
| 26 | O38:H26 | 10 | 1c | 2b | - | + | - |
| 26 | O146:H21 | 829 | - | 2b | - | - | - |
| 27 | O128:H2 | 25 | 1c | - | - | + | - |
| 27 | O130:H11 | 297 | - | 2a | - | + | - |
| 28 | O15:H16 | 325 | - | 2g | - | + | + |
| 28 | O38:H26 | 10 | 1c | 2b | - | + | - |
| 29 | O174:H8 | 13 | 1c | - | - | + | - |
| 29 | O128:H2 | 811 | 1c | 2b | - | - | - |
| 30 | O149:H2 | 388 | - | 2b | - | + | - |
| 30 | O128:H2 | 4748 | 1c | 2b | - | - | - |
| 31 | ONT:H14 |  | - | + | - | - |  |
| 31 | ONT:H2 |  | + | + | - | - |  |
| 32 | O128:H2 |  | - | + | - | - |  |
| 32 | ONT:H7 |  | + | + | + | + |  |
| 33 | O38:H26 |  | + | - | - | + |  |
| 33 | ONT:H7 |  | + | - | - | - |  |
| 34 | O157:H7 | 11 | 1a | 2a | + | + | - |
| 34 | O123:H2 |  | + | - | + | + |  |
| 35 | O157:H7 | 11 | 1a | - | + | + | - |
| 35 | O26:H11 |  | + | - | + | + |  |
| 36 | O38:H26 |  | + | + | - | + |  |
| 36 | ONT:H8 |  | - | + | - | + |  |
| 37 | O157:H7 | 11 | - | 2c | + | + | - |
| 37 | O103:H2 |  | - | + | + | + |  |
| 38 | O163:H19 |  | - | + | - | + |  |
| 38 | ONT:HNM |  | + | - | - | + |  |
| 39 | O157:H7 | 11 | - | 2c | + | + | - |
| 39 | O26:H11 |  | + | - | + | + |  |
| 40 | O157:H7 | 11 | - | 2c | + | + | - |
| 40 | O26:H11 |  | + | - | + | + |  |
| 41 | O157:H7 | 11 | 1a | 2a | + | + | - |
| 41 | ONT:HNM |  | - | + | - | + |  |
| 42 | O26:H11 | 21 | 1a | - | + | + | - |
| 42 | O177:H25 | 342 | - | 2c | + | + | - |
| 43 | O84:H2 | 306 | 1a | - | + | + | - |
| 43 | ONT:H14 |  | - | + | - | - |  |
| 44 | O128:H2 |  | + | + | - | + |  |
| 44 | O38:H26 |  | + | - | - | + |  |
| 45 | O26:H11 | 21 | 1a | - | + | + | - |
| 45 | O157:H7 |  | + | + | + | + |  |

*As not all isolates underwent WGS analysis the phenotypic serotype may be inconclusive, and seven gene MLST and *stx* subtypes may not be displayed as are unknown. NT = not typable, NM = non motile.

Supplementary Table 2. ST Groups assigned for case isolates from 2016-2022 which were subjected to whole genome sequencing analysis and showing comparative EnteroBase assignments (Zhou et al., 2020; Achtman et al., 2022; Dyer et al., 2024).

| ST Group | Serotypes and ST included in Group | EnteroBase ST Complex | EnteroBase HC1100 (cgST Complex) |
| --- | --- | --- | --- |
| ST11 Group  (n=1,193) | O157:H7 ST11 (n= 1,166), ST2966 (n=2), ST10084 (n=9), ST10085 (n=1), ST10420 (n=1), ST10437 (n=2), 10471 (n=1), ST11686, (n=1) ST11798 (n=1), ST11864 (n=4), ST12231 (n=1), ST12826 (n=1), ST13173 (n=1), ST13175 (n=1), ST13518 (n=1) | ST11 Cplx | cgST Cplx 63 |
| ST21 Group  (n=498) | O26:H11 ST21 (n=487), ST8199 (n=1), ST10083 (n=2), ST12853 (n=1)  O111:H8 ST16 (n=3), ST8322 (n=1), ST9345 (n=1)  O103:H8 ST2836 (n=1)  O123:H11 ST29 (n=1) | ST29 Cplx | cgST Cplx 2 |
| ST25 Group  (n=251) | O128:H2 ST25 (n=33), ST811 (n=68), ST4748 (n=89), ST8381 (n=42), ST8422 (n=1), ST10398 (n=1), ST10542 (n=3), ST10653 (n=1), ST10839 (n=1), ST11178 (n=1), ST11305 (n=1), 11349 (n=2), 11486 (n=1), ST11673 (n=1), ST13090 (n=1), ST13196 (n=1), ST13235 (n=1), ST13387 (n=1), STNT* (n=2) | Not assigned | cgST Cplx 1811 |
| ST442 Group  (n=119) | O146:H21 ST442 (n=3), ST829 (n=84), ST10436 (n=3), ST10438 (n=6), ST12507 (n=2)  O174:H21 ST677 (n=3), ST6639 (n=11)  O91:H21 (ST442 (n=5), ST10469 (n=2) | Not assigned | cgST Cplx 877 |
| ST10 Group  (n=110) | O15:H4 ST10 (n=1),  O38:H26 ST10 (n=102), ST11334 (n=1), STNT* (n=1)  O65:H2 ST10 (n=1)  O113:4 ST10 (n=2)  O6:H10 ST43 (n=1)  Onovel32:H10 ST10 (n=1) | ST10 Cplx | cgST Cplx 13 |
| ST17 Group  (n=98) | O15:H2, ST17 (n=7), ST20 (n=2)  O45:H2 ST17 (n=1)  O71:H2 ST17 (n=1)  O103:H2 ST17 (n=38) ST8293 (n=1), ST11862 (n=1), ST12437 (n=2)  O111:H2 ST17 (n=2)  O118:H2 ST17 (n=1)  O123:H2 ST17 (n=5)  O145:H2 ST17 (n=5)  O153:H2 ST17 (n=31)  O177:H2 ST17 (n=1) | ST20 Cplx | cgST Cplx 3 |
| ST300 Group  (n=87) | O84:H2 ST306 (n=35)  O8:H16 ST306 (n=5)  O45:H19 ST306 (n=1)  O159:H4 ST306 (n=2)  O156:H25 ST300 (n=2)  O182:H25 ST300 (18)  O103:H25 ST343 (n=24) | Not assigned | cgST Cplx 60 |
| ST33 Group  (n=73) | O91:H14 ST33 (n=68), O91:HNT# ST33 (n=1), ST13174 (n=1) ST13522 (n=1), STNT* (n=1)  O7:H14 ST33 (n=1) | Not assigned | cgST Cplx 1966 |
| ST57 Group  (n=64) | O176:H4 ST57 (n=60), ST10416 (n=1), ST11863 (n=1), ST12651 (n=1), ST13121 (n=1) | ST350 Cplx | cgST Cplx 175 |
| ST342 Group  (n=55) | O5:HNT# ST342 (n=45)  O177:H25 ST342 (n=6), ST10676 (n=4)  **BUT NOT** O177:H25 ST659 (n=3) | Not assigned | cgST Cplx 836 |
| ST13 Group  (n=50) | O174:H8 ST13 (n=34), ST8630 (n=1), ST10423 (n=6), (ST11333 (n=1), ST11366 (n=1), ST13470 (n=1)  O75:H8 ST13 (n=5), ST10439 (n=1) | ST13 Cplx | cgST Cplx 145 |
| ST297 Group  (n=31) | O130:H11 ST297 (n=27)  O93:H46 ST297 (n=1)  O179:H8 ST297 (n=2), ST9860 (n=1) | Not assigned | cgST Cplx 1081 |
| ST446 Group  (n= 30) | O88:H8 ST446 (n=26), ST11600 (n=1), ST11998 (n=2), ST13019 (n=1) | ST446 Cplx | cgST Cplx 80 |
| ST1308 Group  (n=29) | O64:H20 ST1308 (n=28), ST13120 (n=1) | ST86 Cplx | cgST Cplx 82 |
| ST3695 Group  (n=25) | O123:H10 ST3695 (n=24, ST14269 (n=1) | Not assigned | cgST Cplx 2722 |
| ST56 Group  (n=18) | O113:H21 ST56 (n=4)  O117:H4 ST56 (n=2)  O153:H21 ST56 (n=1)  O100/O154:H25 ST58 (n=3)  066:H25 ST971 (n=2)  O8,O30:H25 ST8660 (n=6) | ST155 Cplx | cgST Cplx 106 |
| ST75 Group (n=18) | O112:H9 ST75 (n=17)  O112:H8 ST75 (n=1) | Not assigned | cgST Cplx 3861 |
| ST679 Group  (n=11) | O163:H19 ST679 (n=11)  **BUT NOT** O163:H19 ST5285 | ST469 Cplx | cgST Cplx 1944 |
| ST504 Group  (n=10) | O117:H7 ST504 (n=4), ST5292 (n=3), ST7641 (n=2), ST10599 (n=1)  **BUT NOT** O117:H7 ST95 | Not assigned | cgST Cplx 1965 |

*STNT: ST not yet assigned

#HNT: H gene coverage insufficient to call H type

Supplementary Table 3. Serotypes, seven gene MLST, and virulence factors associated with the nine NZ STEC case isolates exhibiting hybrid pathogenicity 2016-2022.

| Year | Serotype | Seven gene MLST | *stx1* | *stx2* | *eae* | *ehxA/hly*A | Heat stable toxin (*st*) |
| --- | --- | --- | --- | --- | --- | --- | --- |
| 2016 | O187:H28 | 200 | - | 2g | - | + | + |
| 2019 | O15:H16 | 325 | - | 2g | - | + | + |
| 2019 | O100:H20 | 10399 | - | 2e | - | - | + |
| 2019 | O148:H7 | 10661 | - | 2g | - | + | + |
| 2020 | O51:H24 (n =2) | 10675 | 1c | 2g | - | + | + |
| 2021 | O187:H52 | 642 | 1c | - | - | - | + |
| 2022 | O3:H12 | 329 | 1a | - | - | + | + |
| 2022 | O100:20 | 2514 | - | 2e | - | - | + |

Achtman, M., Zhou, Z., Charlesworth, J., and Baxter, L. (2022). EnteroBase: hierarchical clustering of 100 000s of bacterial genomes into species/subspecies and populations. *Philosophical Transactions of the Royal Society B: Biological Sciences* 377(1861)**,** 20210240. doi: doi:10.1098/rstb.2021.0240.

Dyer, Nigel P., Päuker, B., Baxter, L., Gupta, A., Bunk, B., Overmann, J., et al. (2024). EnteroBase in 2025: exploring the genomic epidemiology of bacterial pathogens. *Nucleic Acids Research*. doi: 10.1093/nar/gkae902.

Zhou, Z., Alikhan, N.F., Mohamed, K., Fan, Y., and Achtman, M. (2020). The EnteroBase user's guide, with case studies on *Salmonella* transmissions, *Yersinia pestis* phylogeny, and *Escherichia* core genomic diversity. *Genome Res* 30(1)**,** 138-152. doi: 10.1101/gr.251678.119.
